# Early identification of Parkinson’s disease with anxiety based on combined clinical and MRI features

**DOI:** 10.3389/fnagi.2024.1414855

**Published:** 2024-06-05

**Authors:** Min Jia, Shijun Yang, Shanshan Li, Siying Chen, Lishuang Wu, Jinlan Li, Hanlin Wang, Congping Wang, Qunhui Liu, Kemei Wu

**Affiliations:** ^1^Department of Neurology, The Central Hospital of Enshi Tujia and Miao Autonomous Prefecture, Enshi, Hubei, China; ^2^Department of Medical Ultrasound, The Central Hospital of Enshi Tujia and Miao Autonomous Prefecture, Enshi, Hubei, China; ^3^Hubei Minzu University, The Central Hospital of Enshi Tujia and Miao Autonomous Prefecture, Enshi, Hubei, China; ^4^Department of Medicine, The Xi’an Jiaotong University, Xi’an, Shanxi, China

**Keywords:** Parkinson’s disease, anxiety, magnetic resonance imaging, clinical features, machine learning

## Abstract

**Objective:**

To identify cortical and subcortical volume, thickness and cortical area features and the networks they constituted related to anxiety in Parkinson’s disease (PD) using structural magnetic resonance imaging (sMRI), and to integrate multimodal features based on machine learning to identify PD-related anxiety.

**Methods:**

A total of 219 patients with PD were retrospectively enrolled in the study. 291 sMRI features including cortical volume, subcortical volume, cortical thickness, and cortical area, as well as 17 clinical features, were extracted. Graph theory analysis was used to explore structural networks. A support vector machine (SVM) combination model, which used both sMRI and clinical features to identify participants with PD-related anxiety, was developed and evaluated. The performance of SVM models were evaluated. The mean impact value (MIV) of the feature importance evaluation algorithm was used to rank the relative importance of sMRI features and clinical features within the model.

**Results:**

17 significant sMRI variables associated with PD-related anxiety was used to build a brain structural network. And seven sMRI and 5 clinical features with statistically significant differences were incorporated into the SVM model. The comprehensive model achieved higher performance than clinical features or sMRI features did alone, with an accuracy of 0.88, a precision of 0.86, a sensitivity of 0.81, an F1-Score of 0.83, a macro-average of 0.85, a weighted-average of 0.92, an AUC of 0.88, and a result of 10-fold cross-validation of 0.91 in test set. The sMRI feature right medialorbitofrontal thickness had the highest impact on the prediction model.

**Conclusion:**

We identified the brain structural features and networks related to anxiety in PD, and developed and internally validated a comprehensive model with multimodal features in identifying.

## 1 Introduction

Parkinson’s disease (PD) is primarily a movement disorder, with the major symptoms including bradykinesia, rigidity, tremors, and postural instability (Kalia and Lang, 2015). However, PD is also often associated with non-motor symptoms, including psychiatric complications, constipation, sensory dysfunctions, cognitive disorders, and sleep disturbances (Špica et al., 2013; Kalia and Lang, 2015; Titova and Chaudhuri, 2017). Anxiety specifically often precedes motor symptoms and is one of the most frequent psychiatric comorbidities in PD, with prevalence estimates ranging from 5.3 to 40% (Broen et al., 2016). Anxiety can accelerate PD progression, aggravate the severity of motor symptoms, alter the therapeutic effectiveness of levodopa, increase pharmacotherapy complications, impact patients’ quality of life, and increase the risk of death (Lintel et al., 2021).

Although previous studies have identified several potential predictors of anxiety in PD (including clinical, serum, imaging, cerebrospinal fluid, neuroelectrophysiological, and neuropsychological factors, as well as PD drug utilization patterns) (Dissanayaka et al., 2010; Zhu et al., 2016; Carey et al., 2020a), there is currently a paucity of comprehensive clinical tools which can early predict the risk of PD-related anxiety. The recent cross-sectional studies and systematic reviews suggest that clinical markers associated with anxiety disorders in PD may include gender, age of disease onset, PD duration, education level, disease stage, motor fluctuations, severity of motor symptoms, cognitive impairment, dose of levodopa, and autonomic symptoms (Zhu et al., 2016; Broen et al., 2018).

Neuroimaging investigations using structural magnetic resonance imaging (sMRI), functional MRI, neurotransmitter/transporter imaging, and metabolic imaging have shown that alterations in the “fear circuit” (composed of the amygdala, anterior cingulate cortex, medial prefrontal cortex, insular cortex, hippocampus, and striatum), as well as “limbic cortico-striatothalamocortical circuits” (involving the prefrontal cortex, basal ganglia, and thalamus) are closely associated with anxiety symptoms (Carey et al., 2020a,b; Sarasso et al., 2020; Carey et al., 2023a,b). sMRI markers from voxel-based morphometry (VBM) and structural covariance analyses have been used to identify cortical markers of anxiety. Specifically, VBM analyses suggest that anxiety is associated with decreased volume in the bilateral anterior cingulate cortices, left amygdala, bilateral precuneus, and bilateral cerebellar tonsils (Vriend et al., 2016; Wee et al., 2016; Oosterwijk et al., 2018; Zhang et al., 2019). In addition, a structural covariance network of the left striatum, right caudate, left striatum, and bilateral prefrontal cortices was negatively correlated with anxiety in PD. Surprisingly, no previous research on PD-related anxiety has concurrently focused on clinical factors and sMRI neuroimaging markers together.

Machine learning algorithms have been widely applied to help identify neuropsychological disorders and guide clinical decision-making (Phokaewvarangkul et al., 2021; Wu et al., 2023). Hilbert et al. (2017) combined clinical questionnaire data, cortisol release data, and structural brain MRI data using a support vector machine (SVM) to separate subjects with generalized anxiety disorder from subjects with depression and healthy controls. Their model had an accuracy of 0.90 for case-classification and an accuracy of 0.67 for disorder-classification. However, there are no studies that have used machine learning to integrate clinical and neuroimaging data to identify PD-related anxiety.

Thus, we analyzed multimodal biobehavioral features, including clinical characteristics, subcortical volume features, cortical volume features, cortical thickness features, and cortical area features, and identified brain regions that were associated with PD-related anxiety. We also used SVM to integrate potential predictors and generate a novel model that could predict cases of PD-related anxiety with high accuracy.

We hypothesized that a certain brain region including subcortical volume, cortical volume, cortical thickness and cortical area, and the networks they make up, were associated with PD-related anxiety, and a comprehensive model could early identify cases of PD-related anxiety.

## 2 Materials and methods

### 2.1 Participants

**Figure 1:**
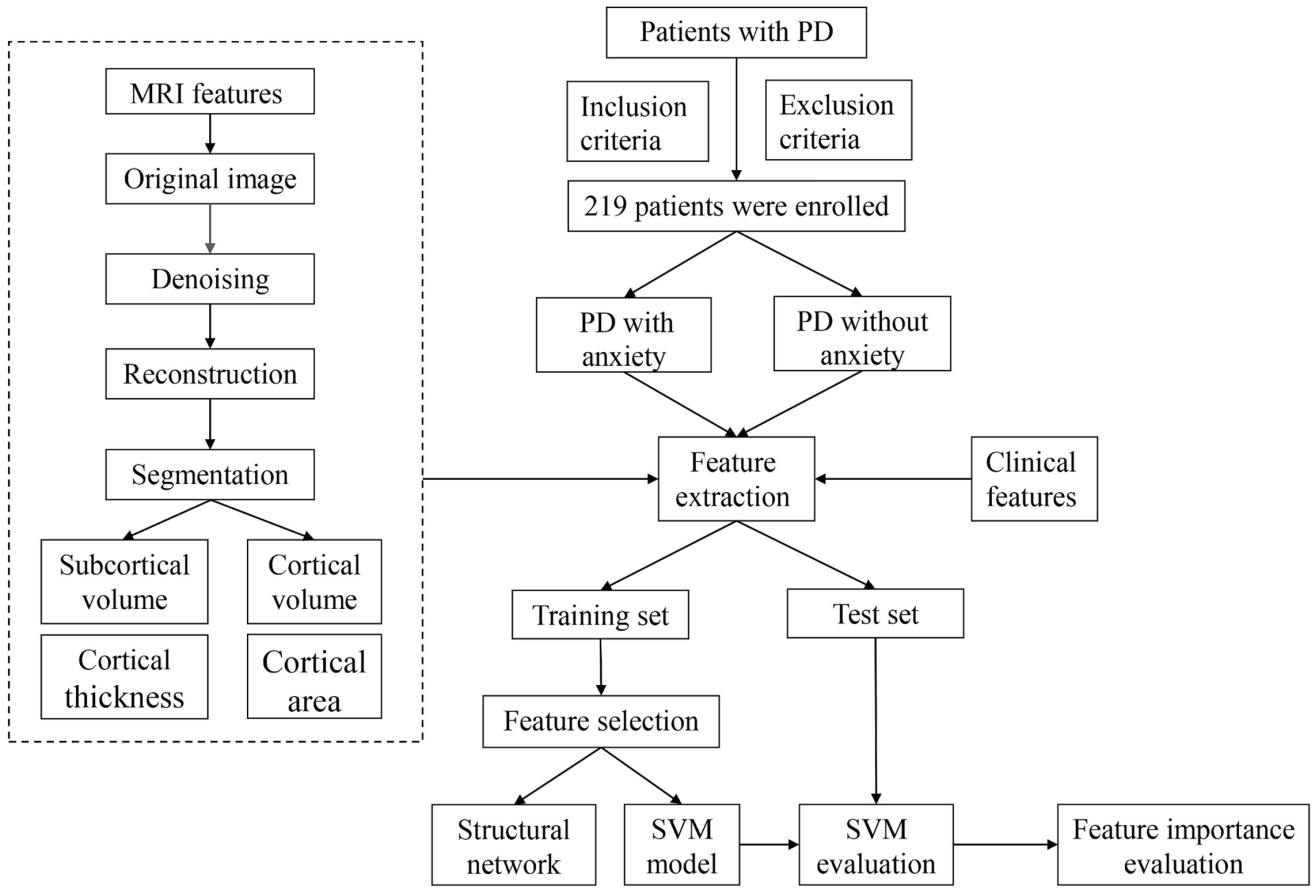
Flow chart. PD, Parkinson’s disease; MRI, magnetic resonance imaging; SVM, support vector machine.

Patients with PD were retrospectively enrolled and were all inpatients at Tujia and Miao Autonomous Prefecture Central Hospital between July 2018 and June 2023. The inclusion criteria were as follows: (1) Patients with PD diagnoses that met UK Brain Bank criteria for idiopathic Parkinson’s disease (Hughes et al., 1992); (2) Patients with completed medical histories and scale evaluation dates; and (3) Patients who underwent neurological examinations and 3D T1 MRI examinations. The exclusion criteria were as follows: (1) Patients with other neurological diseases, such as epilepsy, strokes, tumors, or multiple sclerosis; (2) Patients with history of brain surgery or head trauma; and (3) Patients who were taking antidepressants, anxiolytics, dopaminergic agents, anticholinergic agents, or other centrally acting drugs. Patients with PD were primitively divided into PD with anxiety and PD without anxiety groups (Figure 1). The study was approved by the Tujia and Miao Autonomous Prefecture Central Hospital Institutional Review Board. All participants gave written informed consent.

### 2.2 Clinical measures

Motor symptoms severity was measured with the Unified Parkinson’s Disease Rating Scale–III scale (UPDRS-III), and the disease stage was measured with the Hoehn and Yahr stage scale (H-Y stage). The Mini-Mental State Examination (MMSE) was used to assess general cognitive functioning and memory impairment. Anxiety was evaluated using the Hamilton Anxiety Rating Scale (HAMA) as suggested by the Movement Disorder Society task force (Leentjens et al., 2008). Activities of daily living were assessed using the Activity of Daily Living (ADL) scale. A total of 17 clinical features were summarized.

### 2.3 MRI acquisition

All MRI scans were acquired using a 3 Tesla MRI scanner (Achieva 3.0, Philips Medical Systems, Best, Netherlands). Three-dimensional T1-weighted MPRAGE sequences were acquired with the following parameters: axial acquisition, TR 7.1 ms, TE 3.3 ms, TI 850 ms, FOV 240 × 240 mm^2^, matrix256 × 256, slice thickness 1 mm, total 180 slices, scan time 5:13.

### 2.4 MRI features extraction

**Figure 2:**
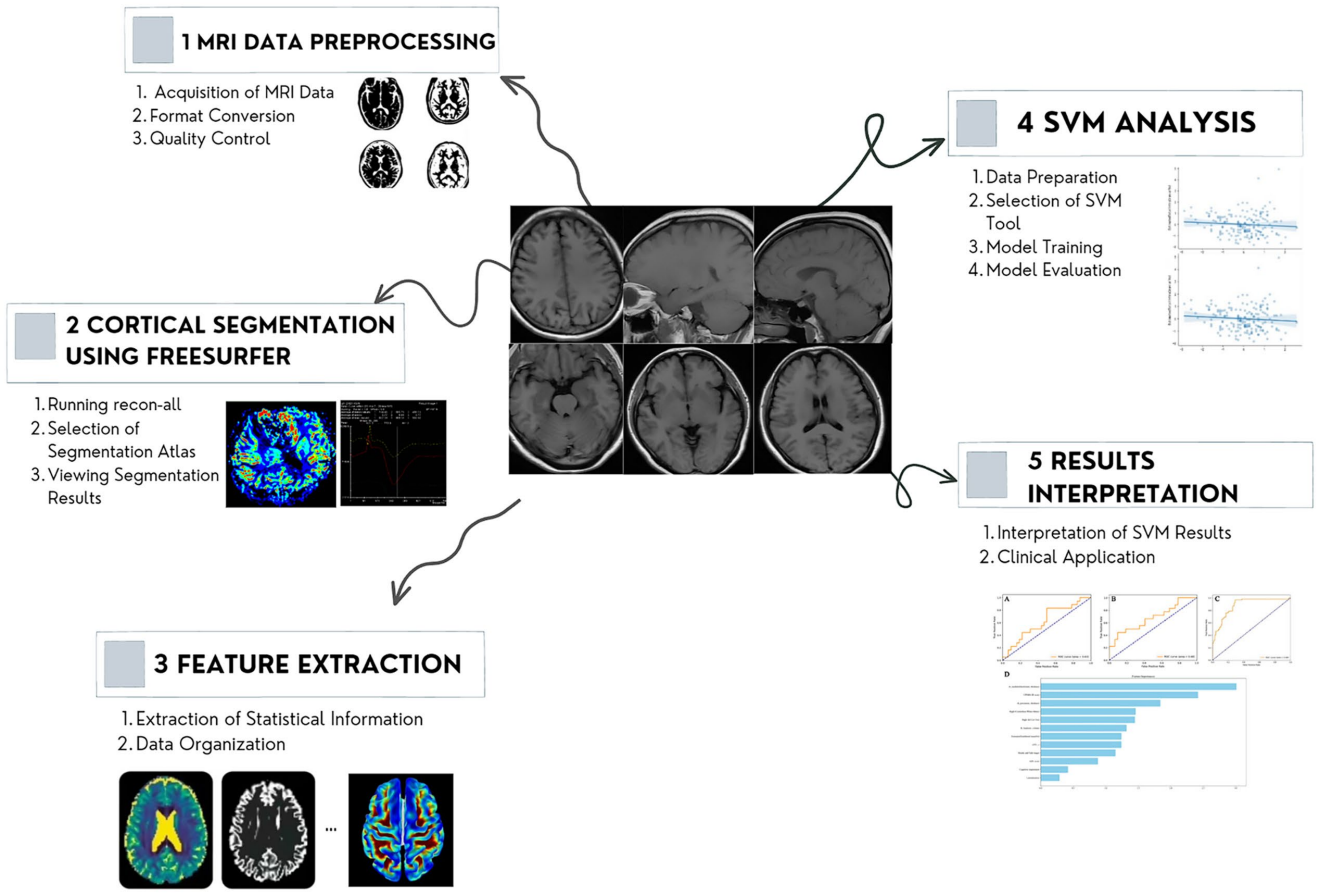
Workflow of the MRI analysis. The proposed method consists of four steps: image acquiring and preprocessing, reconstruction, segmentation, and features extraction. MRI, magnetic resonance imaging.

T1-weighted images in the DICOM format were collected, and FreeSurfer software (Laboratory for Computational Neuroimaging at the Athinoula A. Martinos Center for Biomedical Imaging, version 7.2.0.) in asegstats2table and aparcstats2table was used for denoising, reconstruction, and segmentation to extract sMRI features. First, a series of corrections and smoothing operations to reduce image noise and deviations of incomplete data was executed on T1-weighted sMRI scans. The cortical and subcortical of regions of bilateral hemispheres were parceled using the Desikan–Killiany atlas map to extract regions of interest (ROIs). During the reconstruction process, skull removal, area, and thickness calculations were performed using convolutional neural networks. The quality control analysis of reconstruction included automatic detection of recon-all processing errors and visual inspection for segmentation, intensity normalization, and skull stripping errors. Finally, segmentation of the cerebral cortex and subcortical structures generated a series of quantitative features, including volume, area, thickness, and morphological characteristics. The “recon-all” instruction was used in image processing (Figure 2) (Vriend et al., 2016; Zhang et al., 2021).

### 2.5 MRI network construction

To further clarify the relationship between cortical and subcortical networks and anxiety in PD, we evaluated anatomical networks using extracted sMRI features as follows. First, extracted sMRI features were regarded as regions of interest (ROIs). Subsequently, to avoid repeating edges and self-loops, we create a set of edges assuming only *p*-values < 0.05 would added to the network and added edges in groups based on *p*-values and files. Finally, we mapped the width of the edge based on the weight of the edge using 1/*p*-value to represent the weight. Values were normalized using the weight divided by the maximum weight value if the weight was too small or too large.

### 2.6 Machine learning model

A linear SVM was constructed to identify patients with co-morbid PD and anxiety. The technological process included feature selection, SVM construction, and model evaluation. 80% of the sample (175 patients) was randomly placed into the training set, with the remaining 20% (44 patients) used as a test set to evaluate model performance.

The training set was used to select features and establish the SVM model. An independent sample *t*-test was performed for all sMRI features to compare the differences between patients with and without anxiety. Features that were still significant after Bonferroni correction and FDR adjustment (that is, still had *p* values < 0.05) were selected to build the SVM model. Python SVC algorithms were used for SVM classification. Our SVM model had three main hyperparameters: the kernel, the regularization parameter C, and the kernel hyperparameter (which represented Q of the polynomial kernel and γ of the RBF kernel).

The SVM mode’s performance was evaluated using accuracy, precision, sensitivity, F1-Score, macro-average, and weighted-average. We also calculated the receiver operating characteristic (ROC) curve and the area under the ROC curve (AUC). The formulas were follows:


$$Accuracy\left(A\right)=\left(\frac{TP+TN}{TP+FN+FP+TN}\right)$$

$$Precision\left(P\right)=\left(\frac{TP}{TP+FP}\right)$$

$$Sensitivity\left(S\right)=\left(\frac{TP}{TP+FN}\right)$$

$$F1-score=\left(\frac{2*P*S}{P+S}\right)$$

$$AUC=\frac{\sum_{ins_{i\in \mathrm{positiveclass}}}rank_{ins_{i}}-\frac{M*\left(M+1\right)}{M}}{M+N}$$

The accuracy (A) means the number of the predicted positive samples of all the samples; precision (P) means the number of the actually positive samples of all the predicted positive samples; sensitivity (S) means the number of predicted positive samples of all the actually positive samples; F1-score is the harmonic average of the accuracy and sensitivity, with a maximum of 1 and a minimum of 0. Where true positive (TP) represents the number of predicted positive samples by both the SVM model and the neurologist; false positive (FP) denotes the number of predicted positive samples of PD-related anxiety by the SVM, but their true labels are negative; true negative (TN) is the number of predicted negative samples by the SVM model and the experts; False positive (FN) denotes the number of predicted negative samples of PD-related anxiety by the SVM, but their true labels are positive. A 10-fold cross-validation was also used to measure the universality and practicability of the model: the all dates were divided into 10 equal parts, where one-fold part was utilized for test set, and the rest of the sample was used to train the SVM model.

Macro-average refers to the arithmetic average of each statistical index value of all categories--that is, macro precision and macro sensitivity. The macro-average formula is as follows:


$$\;Macro-Average=\left(\frac{2*Macro-P*Macro-S}{Macro-P+Macro-S}\right)$$

The weighted average is a weighted arithmetic mean of several past observations of the same variable arranged in chronological order and weighted by the number of occurrences of the chronological variable, including weighted precision and weighted sensitivity. The weighted average formula is as follows:


$$Weighted-Average=\left(\frac{2*Weighted-P*Weighted-S}{Weighted-P+Weighted-S}\right)$$

### 2.7 Feature importance evaluation

The mean impact value (MIV) algorithm is considered to be one of the best indexes to assess the relative contribution of candidate features to a given model (Jiang et al., 2013). Thus, we adopted the MIV to evaluate the effect size and significance of different features in our SVM model. When training the final SVM model, which included clinical features and sMRI features, two new training sets were obtained with each independent variable either increased or decreased by 10%, and then used for simulation. Next, the mean of the difference values of the two simulation results was calculated according to the number of samples (thus generating that variable’s MIV). Finally, the independent variables were sorted according to their absolute MIVs, and those on top were identified as potential lead constituents (Jiang et al., 2013; Zhang et al., 2021).

### 2.8 Statistical analysis

The “scipy.stats” package in Python 3.12 was used for statistical analysis of demographic and clinical characteristics. Normality tests were performed on the quantitative data. Means ± standard deviations and *t*-tests were calculated if data were normally distributed; medians, quartiles, and Mann–Whitney U tests were calculated if data were not normally distributed. Qualitative variables were expressed as frequencies and percentages, and Chi-square tests were used to compare differences. 95% confidence intervals (CIs) were also calculated. For all measures, *p*-values<0.05 were considered statistically significant.

## 3 Results

### 3.1 Clinical characteristics

A total of 219 consecutive patients were enrolled in this study, 137 with PD without anxiety and the other 82 with PD-related anxiety, according to their score on the HAMA. Patients were considered “PD-related anxiety” if they had a score above 14 points of HAMA. 175 patients, including 140 patients from the PD without anxiety group and 35 patients from the PD with anxiety group, were randomly divided into a training set, and the remaining 44 participants were used as a test set. Detailed demographic and clinical data for all participants are shown in Table 1.

**Table 1:** The clinical and demographic characteristics in our study.

Variable	PD without anxiety *N* = 137	PD with anxiety *N* = 82	*p*
Age (y)	65.25 ± 9.47	66.62 ± 9.15	0.21
Age of onset (y)	62.75 ± 7.84	57.26 ± 7.95	0.32
Gender			0.89
Male	61 (44.53%)	35 (42.68%)	
Female	76 (55.47%)	47 (51.32%)	
Duration of PD (y)	3.43 ± 2.35	12.10 ± 6.18	0.14
Hypertension			0.77
Yes	50 (36.50%)	28 (34.15%)	
No	87 (63.50%)	54 (65.85%)	
Diabetes			0.17
Yes	11 (8.03%)	12 (14.63%)	
No	126 (91.97%)	70 (85.37%)	
Smoking			0.86
Yes	27 (%)	15 (18.29%)	
No	110 (%)	67 (81.71%)	
Drinking			0.70
Yes	19 (13.87%)	13 (15.85%)	
No	118 (86.13%)	69 (84.15%)	
Tea			0.60
Yes	9 (6.57%)	7 (8.54%)	
No	128 (93.43%)	75 (91.46%)	
Cognitive impairment			0.03
Yes	4 (2.92%)	6 (7.32%)	
No	133 (97.08%)	76 (92.68%)	
History of anxiety and depression			0.80
Yes	10 (%)	7 (8.54%)	
No	127 (%)	75 (91.46%)	
Daily levodopa dose, mg	570 ± 125	660 ± 125	0.31
Symptom onset side			<0.00
Left	31 (22.63%)	9 (10.98%)	
Right	36 (26.28%)	12 (14.63%)	
Both	70 (51.09%)	61 (74.39%)	
Hoehn and Yahr stages			<0.00
Mild (1, 1.5, and 2)	64 (46.71%)	21 (25.61%)	
Moderate (2.5 and 3)	44 (32.12%)	37 (45.12%)	
Severe (4 and 5)	29 (21.17%)	24 (29.27%)	
ADL score	90 ± 5	80 ± 5	0.02
UPDRS-III score	8 ± 1.5	11 ± 3	0.02
Education in years	6 ± 1.8	6 ± 2.3	0.57

PD, Parkinson’s disease; ADL, activity of daily living scale; UPDRS-III, unified Parkinson’s disease rating scale–III; *p*, *p* value. *p* < 0.05 was considered to be significant.

### 3.2 Features extraction and selection

In the training set, a total of 291 sMRI features related to cortical thickness, cortical area, cortical volume, and subcortical volume were extracted from each patient (Appendix 1). This included four classes of radiomics features: (a) 65 subcortical volume features, (b) 74 cortical volume features, (c) 76 cortical thickness features, and (d) and 76 cortical area features. Of the 291 features, seven candidate predictors were incorporated into the SVM model: “Right medialorbitofrontal thickness”, “Right precuneus thickness,” “Right cerebellum white matter,” “Right inferior lateral ventricle”, “Left bankssts volume,” “Estimate total lumen volume”, and “Estimated total intracranial volume.”

### 3.3 sMRI network

**Figure 3:**
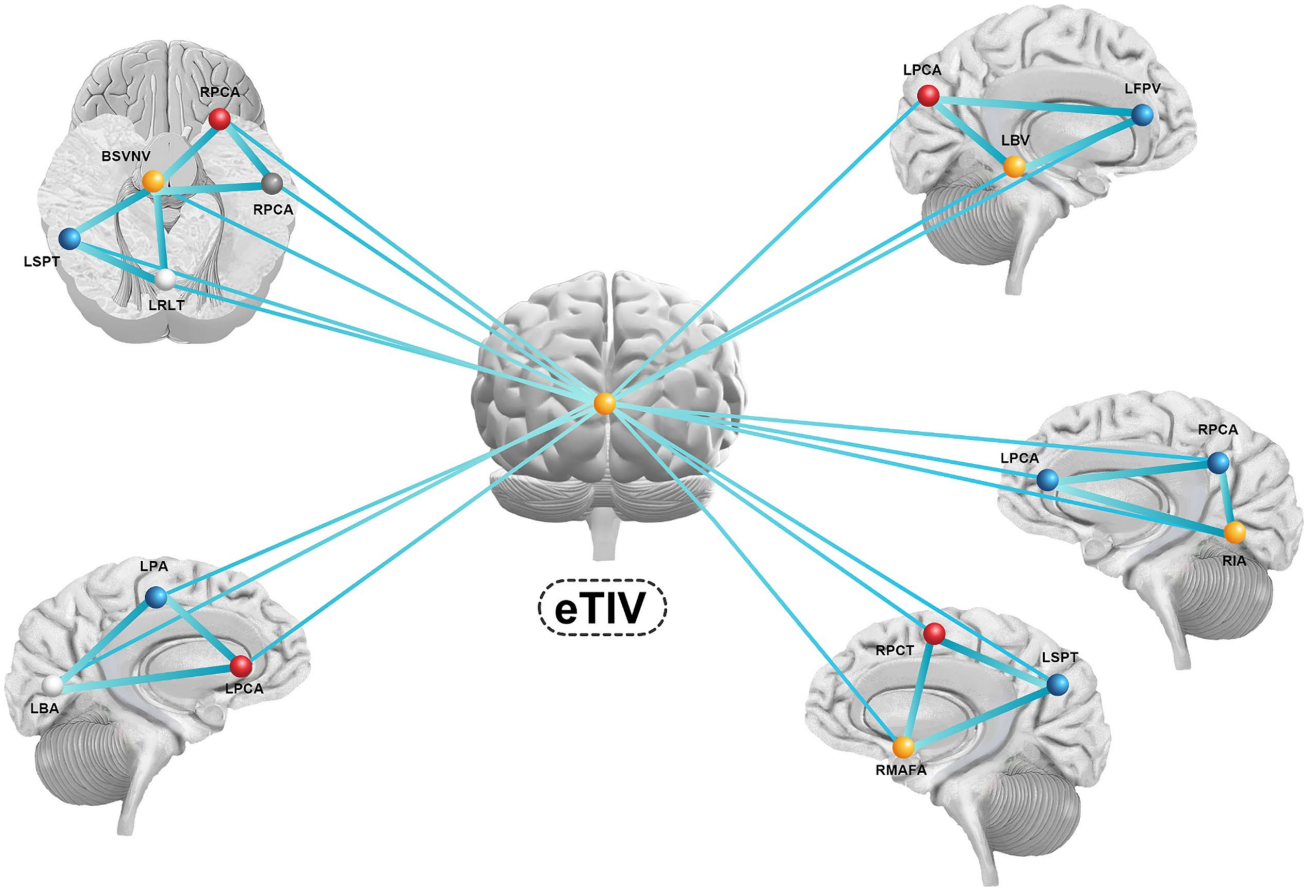
The features and network structure for a schematic representation of the results. eTIV, estimate total lumen volume; RPCA, right postcentral area; RPCA, right precentral area; BSVNV, brain segmentation volume not ventricle; RPCA, right postcentral area; RMAFA, right medialorbitofrontal area; RIA, right insula area; RPCT, right precuneus thickness; RSTT, right superiortemporal thickness; RMBFT, right medialorbitofrontal thickness; LSPT, light superiorparietal thickness; LRLT, light rostralanteriorcingulate thickness; LPA, light parsorbitalis area; LBA, light bankssts area; LPCA, light precentral area; LPBV, light parsorbitalis volume; LFPV, light frontalpole volume; LBV, light bankssts volume.

We selected significant sMRI variables and then crafted a structural brain network associated with PD-related anxiety based on graph theory. 17 ROIs in the network were positively correlated with PD-related anxiety. Figure 3 shows the features and network structure for a schematic representation of the results (Figure 3).

### 3.4 Machine learning model

**Figure 4:**
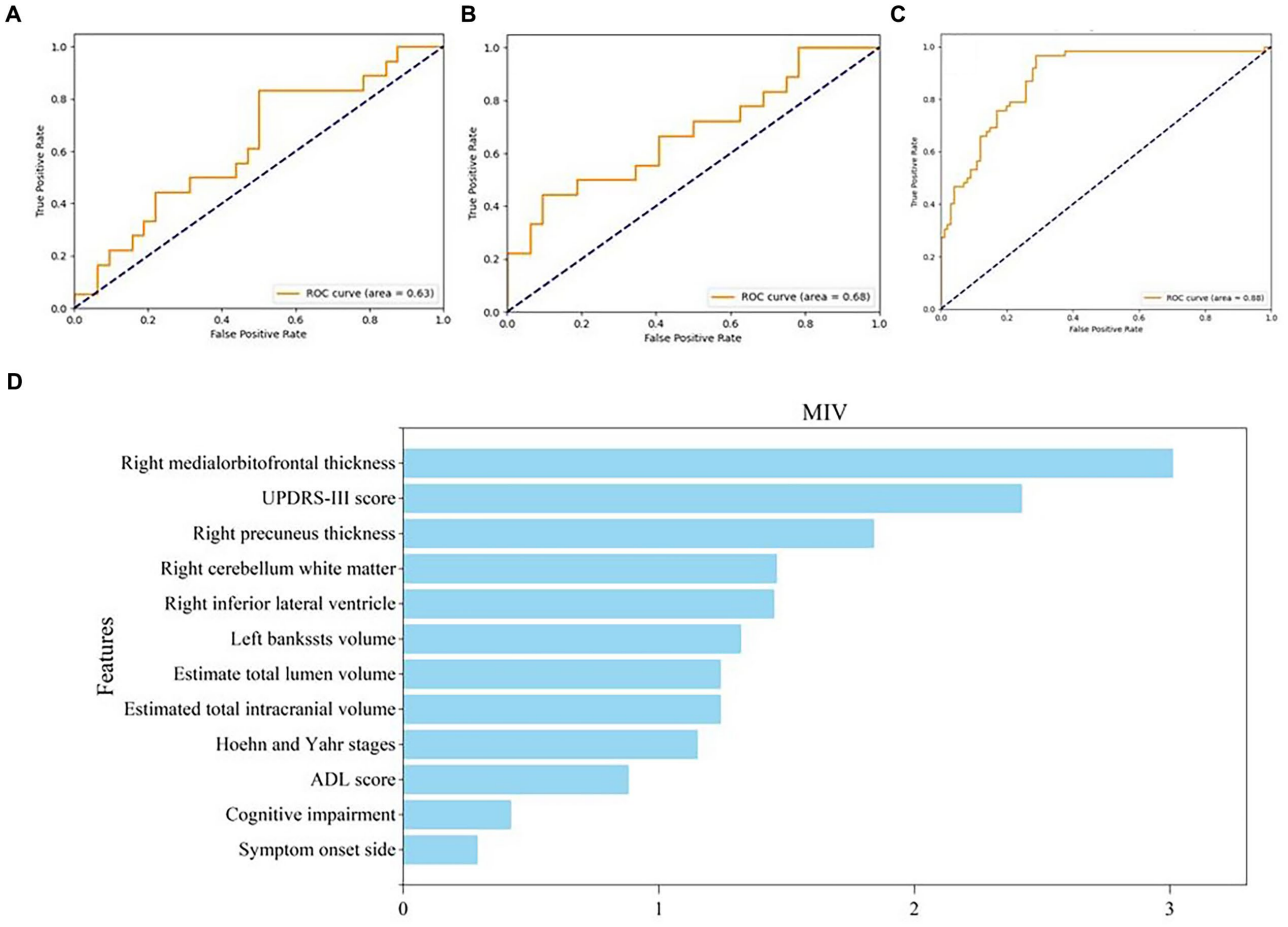
ROC curve of three models and feature importance evaluation of clinical and MRI features. **(A–C)** The ROC curve of clinical model, MRI model and clinical-MRI model, respectively; **(D)** The feature importance evaluation of clinical and MRI features. ROC, receiver operating characteristic; MRI, magnetic resonance imaging.

We built three SVM models to identify subjects with anxiety in PD. Model 1 (the clinical model) was only based on clinical characteristics, model 2 (the sMRI model) was only based on sMRI characteristics, and model 3 (the clinical-sMRI model) was an integrative prediction algorithm based on combined clinical-sMRI features. The comprehensive model achieved higher performance in identifying patients with PD-related anxiety than either the clinical features or sMRI features models, with an accuracy of 0.88, a precision of 0.86, a sensitivity of 0.81, an F1-Score of 0.83, a macro-average of 0.85, a weighted-average 0.92, an AUC of 0.88, and a result of 10-fold cross-validation of 0.91 in the test set (Table 2). Figure 4 displays the ROC curve. The feature importance sequence showed that the right medialorbitofrontal thickness feature played the most important role in the SVM model. These results are summarized in Table 3 and Figure 4.

**Table 2:** The performance of three models for identifying PD-related anxiety.

	Group	AC	PR	SE	F1-score	MA	WA	AUC	*k*-fold validation
Clinical model	Train	0.68	0.71	0.89	0.75	0.47	0.66	0.65	0.71
	Test	0.64	0.64	1.00	0.78	0.50	0.64	0.63	
MRI model	Train	0.70	0.74	0.88	0.85	0.80	0.70	0.70	0.80
	Test	0.74	0.74	0.91	0.82	0.74	0.72	0.68	
Clinical-MRI model	Train	0.85	0.88	0.78	0.85	0.85	0.92	0.87	0.91
	Test	0.88	0.86	0.81	0.83	0.85	0.92	0.88	

PD, Parkinson’s disease; MRI, magnetic resonance imaging; AC, accuracy; PR, precision; SE, sensitivity; MA, macro-average; WA, weighted-average; AUC, area under the curve.

**Table 3:** The feature importance sequence of MIV.

Ranking	Features	MIV
1	Right medialorbitofrontal thickness	3.01
2	UPDRS-III score	2.42
3	Right precuneus thickness	1.84
4	Right cerebellum white matter	1.46
5	Right inferior lateral ventricle	1.45
6	Left bankssts volume	1.32
7	Estimate total lumen volume	1.24
8	Estimated total intracranial volume	1.24
9	Hoehn and Yahr stages	1.15
10	ADL score	0.88
11	Cognitive impairment	0.42
12	Symptom onset side	0.29

MIV, mean impact value; UPDRS-III, unified Parkinson’s disease rating scale–III; ADL, activity of daily living scale.

## 4 Discussion

In this study, we extracted whole brain sMRI features, including subcortical volume, cortical volume, cortical thickness, and cortical area features, and localized abnormal brain regions and anxiety network structures in patients with PD-related anxiety. We also combined these sMRI features with clinical features to establish an SVM model which could identify patients with PD-related anxiety with higher accuracy and precision than models based on either clinical features or sMRI features alone. Thus, our mixed model performed very well and may eventually be suitable for clinical application.

Many studies have explored the relationship between clinical symptoms and anxiety in patients with PD. Previous studies have shown that the severity of PD, the duration of PD, PD with postural instability and gait dysfunction symptom clustering, high levodopa dosage, experiences of dyskinesias or on/off fluctuations, lateralisation of PD, low ADL functionality, and comorbidities such as diabetes, may be potential risk factors for co-morbid anxiety in PD (Dissanayaka et al., 2010; Noyce et al., 2012; Sagna et al., 2014; Wee et al., 2016; Zhu et al., 2016; Cui et al., 2017). Sagna et al. (2014) performed a systematic review of factors associated with anxiety and depression in PD and found that several clinical factors, including autonomic symptoms, motor fluctuations, severity and frequency of symptoms, staging of disease, and PD onset and duration, were associated with anxiety and depression among older adults suffering from PD. Cui et al. (2017) explored the relationship between anxiety and PD in Chinese patients and found that female gender, higher rapid eye movement behavior disorder Questionnaire-Hong Kong (RBD-HK) scores, higher Hamilton Depression Rating Scale (HAMD) scores, autonomic symptoms, and larger substantia nigra echogenic areas were associated with anxiety. However, there are still major challenges associated with identifying co-morbid PD and anxiety using only clinical features.

Neuroimaging, including anatomical MRI, fMRI, neurotransmitter/transporter imaging, and metabolic imaging, has played an irreplaceable role in identifying the brain regions and circuits involved in PD-related anxiety (Carey et al., 2020a). sMRI is the most practical of these approaches in clinical settings. Wee et al. (2016) conducted a prospective longitudinal VBM study and found that anxiety symptoms were associated with decreased grey matter volumes in the bilateral precuneus and anterior cingulate cortex. Vriend et al. (2016) found that decreases in amygdala volume constituted a premorbid trait also using VBM. Using the left amygdala as a seed, Zhang et al. (2019) further explored cortical correlations of anxiety and found that it was positively correlated with the functional connectivity between the amygdala and the superior parietal lobule using voxel-based neuroanatomical and functional connectivity measures. Another study investigated the pathophysiology of anxiety in PD using structural covariance and found that anxiety was correlated with lower interstriatal and striatal-prefrontal connectivity (Oosterwijk et al., 2018).

Electrophysiology, including electroencephalograms and electromyography, can also reflect motor symptom severity and pharmaceutical effects in PD patients (Waninger et al., 2020; Betrouni et al., 2022). Previous studies have also shown that saliva, urine, blood, and cerebrospinal fluid (CSF) biomarkers can help identify patients with PD and aid in disease monitoring and subtype characterization (Maass et al., 2019; Cocco et al., 2023; Xiang et al., 2024). However, whether these markers can be used to identify cases of co-morbid PD and anxiety needs further study. Theoretically, we could build a comprehensive model of PD and anxiety that would integrate clinical features, electrophysiology features, radiomics (including MRI, fMRI, DTI, PET-CT, PET-MRI, etc.), genomic features, and proteomic features. However, clinical and sMRI features are the most readily available in clinical practice and have both already been shown to be closely related to PD-related anxiety.

Selecting effective variables when building a model can help avoid overfitting and increase a model’s accuracy and clinical application value. Here, we first selected useful sMRI variables and established network connections based on graph theory methods. We next used a statistical approach to select potential features to establish SVM models. We used two different analysis techniques to select features and demonstrated the accuracy of the variables that we selected.

Although many approaches have been developed to aid in the early diagnosis, treatment monitoring, subtype characterization, and identification of cognitive impairment among PD patients, few studies have combined clinical features and MRI features to create models to predict the occurrence of PD-related anxiety. In our study, a combined clinical-MRI features model showed stronger potential to differentiate between PD patients with anxiety and PD patients without anxiety compared with single-modality models. Although new models related to the occurrence of anxiety in PD may be developed in the future, the performance and populations will likely vary. Thus, more studies on modeling are needed and could be used to compare with our findings.

sMRI compared with serum markers or electrophysiology is the most common non-invasive, convenient, and safe examination in clinical settings. We demonstrated that an SVM model that combined clinical and sMRI features had improved prediction accuracy and clinical practicability. However, our study has some limitations. First, the sample size and population were limited, which may have led to bias and clinical heterogeneity. Thus, our results need to be replicated with larger, prospective, multicenter samples, and externally validated. Second, the anxiety was evaluated using the Hamilton Anxiety Rating Scale (HAMA) as suggested by the Movement Disorder Society task force in 2008, and the Parkinson’s Anxiety Scale (PAS) should be used in future research. Third, we only included thickness, volume, and area sMRI characteristics. Multimodal images could be included in the future, such as iron charge deposition, shape or texture of structures. Finally, electrophysiological and serum or cerebrospinal fluid biomarkers of PD-related anxiety should be explored and included in future predictive models.

## 5 Conclusion

In this study, a comprehensive analysis of sMRI features suggested that PD-related anxiety was associated with specific cortical and subcortical features and network structures. A comprehensive SVM model with multimodal features was also developed and internally validated and found to have excellent accuracy and clinical practicability for identifying cases of co-morbid anxiety and PD.

## Data availability statement

The datasets presented in this article are not readily available because of the requirement of the Ethics Committee of Tujia and Miao Autonomous Prefecture Central Hospital (Enshi, China). Requests to access the datasets should be directed to the corresponding author.

## Ethics statement

The studies involving humans were approved by the Tujia and Miao Autonomous Prefecture Central Hospital Institutional Review Board. The studies were conducted in accordance with the local legislation and institutional requirements. Written informed consent for participation in this study was provided by the participants’ legal guardians/next of kin.

## Author contributions

MJ: Project administration, Resources, Visualization, Writing – original draft, Writing – review & editing. SY: Conceptualization, Methodology, Software, Writing – original draft. SL: Formal analysis, Methodology, Visualization, Writing – review & editing. SC: Writing – original draft, Data curation, Validation. LW: Data curation, Writing – review & editing. JL: Funding acquisition, Resources, Writing – review & editing. HW: Data curation, Writing – review & editing. CW: Writing – review & editing. QL: Writing – review & editing. KW: Project administration, Writing – review & editing.
