# Supplementary material for: Early identification of Parkinson’s disease with anxiety based on combined clinical and MRI features
Source: Front Aging Neurosci. 2024 Jun 5;16:1414855. doi: 10.3389/fnagi.2024.1414855 (PMC11188332; doi:10.3389/fnagi.2024.1414855)
Supplement: Supplementary file 1 [file Data_Sheet_1.docx]

Appendix 1 The 291 structural magnetic resonance imaging features.

{"aseg_volume_table": [

"Measure:volume",

"Left-Lateral-Ventricle",

"Left-Inf-Lat-Vent",

"Left-Cerebellum-White-Matter",

"Left-Cerebellum-Cortex",

"Left-Thalamus",

"Left-Caudate",

"Left-Putamen",

"Left-Pallidum",

"3rd-Ventricle",

"4th-Ventricle",

"Brain-Stem",

"Left-Hippocampus",

"Left-Amygdala",

"CSF",

"Left-Accumbens-area",

"Left-VentralDC",

"Left-vessel",

"Left-choroid-plexus",

"Right-Lateral-Ventricle",

"Right-Inf-Lat-Vent",

"Right-Cerebellum-White-Matter",

"Right-Cerebellum-Cortex",

"Right-Thalamus",

"Right-Caudate",

"Right-Putamen",

"Right-Pallidum",

"Right-Hippocampus",

"Right-Amygdala",

"Right-Accumbens-area",

"Right-VentralDC",

"Right-vessel",

"Right-choroid-plexus",

"5th-Ventricle",

"WM-hypointensities",

"Left-WM-hypointensities",

"Right-WM-hypointensities",

"non-WM-hypointensities",

"Left-non-WM-hypointensities",

"Right-non-WM-hypointensities",

"Optic-Chiasm",

"CC_Posterior",

"CC_Mid_Posterior",

"CC_Central",

"CC_Mid_Anterior",

"CC_Anterior",

"BrainSegVol",

"BrainSegVolNotVent",

"lhCortexVol",

"rhCortexVol",

"CortexVol",

"lhCerebralWhiteMatterVol",

"rhCerebralWhiteMatterVol",

"CerebralWhiteMatterVol",

"SubCortGrayVol",

"TotalGrayVol",

"SupraTentorialVol",

"SupraTentorialVolNotVent",

"MaskVol",

"BrainSegVol-to-eTIV",

"MaskVol-to-eTIV",

"lhSurfaceHoles",

"rhSurfaceHoles",

"SurfaceHoles",

"EstimatedTotalIntraCranialVol"],

　　"lh_aparc_volume_table": [

"lh.aparc.volume",

"lh_bankssts_volume",

"lh_caudalanteriorcingulate_volume",

"lh_caudalmiddlefrontal_volume",

"lh_cuneus_volume",

"lh_entorhinal_volume",

"lh_fusiform_volume",

"lh_inferiorparietal_volume",

"lh_inferiortemporal_volume",

"lh_isthmuscingulate_volume",

"lh_lateraloccipital_volume",

"lh_lateralorbitofrontal_volume",

"lh_lingual_volume",

"lh_medialorbitofrontal_volume",

"lh_middletemporal_volume",

"lh_parahippocampal_volume",

"lh_paracentral_volume",

"lh_parsopercularis_volume",

"lh_parsorbitalis_volume",

"lh_parstriangularis_volume",

"lh_pericalcarine_volume",

"lh_postcentral_volume",

"lh_posteriorcingulate_volume",

"lh_precentral_volume",

"lh_precuneus_volume",

"lh_rostralanteriorcingulate_volume",

"lh_rostralmiddlefrontal_volume",

"lh_superiorfrontal_volume",

"lh_superiorparietal_volume",

"lh_superiortemporal_volume",

"lh_supramarginal_volume",

"lh_frontalpole_volume",

"lh_temporalpole_volume",

"lh_transversetemporal_volume",

"lh_insula_volume",

"BrainSegVolNotVent",

"eTIV" ],

　　"rh_aparc_volume_table": [

"rh.aparc.volume",

"rh_bankssts_volume",

"rh_caudalanteriorcingulate_volume",

"rh_caudalmiddlefrontal_volume",

"rh_cuneus_volume",

"rh_entorhinal_volume",

"rh_fusiform_volume",

"rh_inferiorparietal_volume",

"rh_inferiortemporal_volume",

"rh_isthmuscingulate_volume",

"rh_lateraloccipital_volume",

"rh_lateralorbitofrontal_volume",

"rh_lingual_volume",

"rh_medialorbitofrontal_volume",

"rh_middletemporal_volume",

"rh_parahippocampal_volume",

"rh_paracentral_volume",

"rh_parsopercularis_volume",

"rh_parsorbitalis_volume",

"rh_parstriangularis_volume",

"rh_pericalcarine_volume",

"rh_postcentral_volume",

"rh_posteriorcingulate_volume",

"rh_precentral_volume",

"rh_precuneus_volume",

"rh_rostralanteriorcingulate_volume",

"rh_rostralmiddlefrontal_volume",

"rh_superiorfrontal_volume",

"rh_superiorparietal_volume",

"rh_superiortemporal_volume",

"rh_supramarginal_volume",

"rh_frontalpole_volume",

"rh_temporalpole_volume",

"rh_transversetemporal_volume",

"rh_insula_volume",

"BrainSegVolNotVent",

"eTIV"]

"lh_aparc_area_table": [

"lh.aparc.area",

"lh_bankssts_area",

"lh_caudalanteriorcingulate_area",

"lh_caudalmiddlefrontal_area",

"lh_cuneus_area",

"lh_entorhinal_area",

"lh_fusiform_area",

"lh_inferiorparietal_area",

"lh_inferiortemporal_area",

"lh_isthmuscingulate_area",

"lh_lateraloccipital_area",

"lh_lateralorbitofrontal_area",

"lh_lingual_area",

"lh_medialorbitofrontal_area",

"lh_middletemporal_area",

"lh_parahippocampal_area",

"lh_paracentral_area",

"lh_parsopercularis_area",

"lh_parsorbitalis_area",

"lh_parstriangularis_area",

"lh_pericalcarine_area",

"lh_postcentral_area",

"lh_posteriorcingulate_area",

"lh_precentral_area",

"lh_precuneus_area",

"lh_rostralanteriorcingulate_area",

"lh_rostralmiddlefrontal_area",

"lh_superiorfrontal_area",

"lh_superiorparietal_area",

"lh_superiortemporal_area",

"lh_supramarginal_area",

"lh_frontalpole_area",

"lh_temporalpole_area",

"lh_transversetemporal_area",

"lh_insula_area",

"lh_WhiteSurfArea_area",

"BrainSegVolNotVent",

"eTIV"],

　　"rh_aparc_area_table": [

"rh.aparc.area",

"rh_bankssts_area",

"rh_caudalanteriorcingulate_area",

"rh_caudalmiddlefrontal_area",

"rh_cuneus_area",

"rh_entorhinal_area",

"rh_fusiform_area",

"rh_inferiorparietal_area",

"rh_inferiortemporal_area",

"rh_isthmuscingulate_area",

"rh_lateraloccipital_area",

"rh_lateralorbitofrontal_area",

"rh_lingual_area",

"rh_medialorbitofrontal_area",

"rh_middletemporal_area",

"rh_parahippocampal_area",

"rh_paracentral_area",

"rh_parsopercularis_area",

"rh_parsorbitalis_area",

"rh_parstriangularis_area",

"rh_pericalcarine_area",

"rh_postcentral_area",

"rh_posteriorcingulate_area",

"rh_precentral_area",

"rh_precuneus_area",

"rh_rostralanteriorcingulate_area",

"rh_rostralmiddlefrontal_area",

"rh_superiorfrontal_area",

"rh_superiorparietal_area",

"rh_superiortemporal_area",

"rh_supramarginal_area",

"rh_frontalpole_area",

"rh_temporalpole_area",

"rh_transversetemporal_area",

"rh_insula_area",

"rh_WhiteSurfArea_area",

"BrainSegVolNotVent",

"eTIV"],

"lh_aparc_thickness_table": [

"lh.aparc.thickness",

"lh_bankssts_thickness",

"lh_caudalanteriorcingulate_thickness",

"lh_caudalmiddlefrontal_thickness",

"lh_cuneus_thickness",

"lh_entorhinal_thickness",

"lh_fusiform_thickness",

"lh_inferiorparietal_thickness",

"lh_inferiortemporal_thickness",

"lh_isthmuscingulate_thickness",

"lh_lateraloccipital_thickness",

"lh_lateralorbitofrontal_thickness",

"lh_lingual_thickness",

"lh_medialorbitofrontal_thickness",

"lh_middletemporal_thickness",

"lh_parahippocampal_thickness",

"lh_paracentral_thickness",

"lh_parsopercularis_thickness",

"lh_parsorbitalis_thickness",

"lh_parstriangularis_thickness",

"lh_pericalcarine_thickness",

"lh_postcentral_thickness",

"lh_posteriorcingulate_thickness",

"lh_precentral_thickness",

"lh_precuneus_thickness",

"lh_rostralanteriorcingulate_thickness",

"lh_rostralmiddlefrontal_thickness",

"lh_superiorfrontal_thickness",

"lh_superiorparietal_thickness",

"lh_superiortemporal_thickness",

"lh_supramarginal_thickness",

"lh_frontalpole_thickness",

"lh_temporalpole_thickness",

"lh_transversetemporal_thickness",

"lh_insula_thickness",

"lh_MeanThickness_thickness",

"BrainSegVolNotVent",

"eTIV"],

"rh_aparc_thickness_table": [

"rh.aparc.thickness",

"rh_bankssts_thickness",

"rh_caudalanteriorcingulate_thickness",

"rh_caudalmiddlefrontal_thickness",

"rh_cuneus_thickness",

"rh_entorhinal_thickness",

"rh_fusiform_thickness",

"rh_inferiorparietal_thickness",

"rh_inferiortemporal_thickness",

"rh_isthmuscingulate_thickness",

"rh_lateraloccipital_thickness",

"rh_lateralorbitofrontal_thickness",

"rh_lingual_thickness",

"rh_medialorbitofrontal_thickness",

"rh_middletemporal_thickness",

"rh_parahippocampal_thickness",

"rh_paracentral_thickness",

"rh_parsopercularis_thickness",

"rh_parsorbitalis_thickness",

"rh_parstriangularis_thickness",

"rh_pericalcarine_thickness",

"rh_postcentral_thickness",

"rh_posteriorcingulate_thickness",

"rh_precentral_thickness",

"rh_precuneus_thickness",

"rh_rostralanteriorcingulate_thickness",

"rh_rostralmiddlefrontal_thickness",

"rh_superiorfrontal_thickness",

"rh_superiorparietal_thickness",

"rh_superiortemporal_thickness",

"rh_supramarginal_thickness",

"rh_frontalpole_thickness",

"rh_temporalpole_thickness",

"rh_transversetemporal_thickness",

"rh_insula_thickness",

"rh_MeanThickness_thickness",

"BrainSegVolNotVent",

"eTIV"],

}
